# Is There a Limit to Resemblances? Teaching About Science and Pseudoscience From a Family Resemblance Perspective

**DOI:** 10.1007/s11191-022-00394-4

**Published:** 2022-10-11

**Authors:** Wonyong Park, Richard Brock

**Affiliations:** 1 University of Southampton, Southampton, UK; 2 King’s College London, London, UK

## Abstract

The notion of family resemblance has recently emerged as a promising and fruitful approach to characterising the nature of science (NOS) in science education research, offering solutions to some perplexing challenges such as capturing both the domain-general and domain-specific features of science with a single framework. At the same time, however, criticism has been levelled that the resemblance might eventually extend to certain activities that are not scientific but pose as science. This would be an undesirable consequence for science educators, particularly given the increasing need for individuals to discern pseudoscientific claims circulated on social media from scientific information. Many pseudoscientific and non-scientific activities resemble science in terms of their aim to explain nature, their use of evidence-based methods, and their interrelation with politics and society. In this theoretical article, we build on the concept of family resemblance to consider how it can simultaneously explain the diversity and unity of science and help students to learn about the nature of science and that of pseudoscience in science education. We put forward three principles that can guide teaching about pseudoscience based on the family resemblance conceptualisation of science.

## What Science is and is Not

In January 2020, the Indian government recommended the use of remedies based on homoeopathy and traditional medicine to ward off the new coronavirus infections. It did not take long for the decision to provoke fierce criticism from doctors and scientists across the world, who argued that it “entirely undermines public understanding of science and medicine and elevates pseudoscience with potentially dangerous consequences” (Dasgupta, 2020, para 2). How do we know homoeopathy is a pseudoscience instead of legitimate science? Gordin (2021) explains that what makes something science rather than pseudoscience is not merely the fact that it produces correct information. This is because, as Popper (1963) wrote, “science often errs, and that pseudoscience may happen to stumble on the truth” (p. 44). Homoeopathy, first conceived by the German physician Samuel Hahnemann at the end of the eighteenth century, postulates that super-diluted natural substances such as plants and minerals will cure symptoms that these substances cause in a healthy person. It has some “theoretical” grounds and there are also institutions to disseminate homoeopathy and train practitioners—The American Institute of Homoeopathy has a slightly longer history than the American Medical Association (American Institute of Homoeopathy, 2007)—in some countries, homoeopathy even receives substantial financial support from the public healthcare system (e.g., Ministry of Health & Family Welfare, 2016). There are some meta-analytical studies, published in peer-reviewed journals, empirically showing homoeopathy’s effectiveness beyond a placebo. The problem, however, is that the empirical evidence favourable to homoeopathy is insufficient and too anecdotal to be accepted as a treatment in models of evidence-based medicine, and theoretical assumptions such as “like cures like” are not coherent with scientific knowledge. That is, homoeopathy is a pseudoscience despite having some science-like features. This case points to the crucial role of understanding the NOS, besides scientific knowledge itself, in discerning trustworthy and reliable information in times of crisis, but more generally, also in our daily lives.

Understanding the meaning and mechanisms of science has become of paramount significance in society today, where “facts [are] uncertain, values [are] in dispute, stakes [are] high and decisions [are] urgent” (Funtowicz & Ravetz, 1993, p. 744). In recent years, family resemblance has become popular as a framework for NOS research in science education. The idea of family resemblance is that various human activities that we think of as “science” cannot be characterised in terms of a set of non-trivial, necessary and jointly sufficient conditions but only understood as a “family” concept (Wittgenstein, 1953). That is, “sciences” can be grouped together by open-ended features that are shared by some members, but not all the features need to be shared amongst all members. Apart from the famous example of “games” used by Wittgenstein himself, scholars in different fields have since capitalised on its explanatory power to provide definitions of abstract concepts such as fine art (Weitz, 1956), religion (Saler, 1999), power (Haugaard, 2010), fascism (Eco, 1995), literary genres (Fishelov, 1991), intellectual property (George, 2012) and entrepreneurship (Leunbach, 2021), which demonstrates the fruitfulness of family resemblances in understanding complex concepts. John Dupré was amongst the first to note the potential of family resemblance in explaining what science is. In *The Disorder of Things* (Dupré, 1993), he recognised the heterogeneity of science and wrote:

Science, to borrow an important idea from the later Wittgenstein, is best seen as a family resemblance concept. That is, there will be a number, perhaps an indefinite number, of features characteristic of parts of science, and every part of science will have some of these features, but very probably none will have all. (p. 242)

The usefulness of the family resemblance approach (FRA) in providing a comprehensive account of “science” as a dynamic and heterogeneous enterprise led to its application in the context of science education. The FRA provides a “polythetic” classification of a concept based on “a complex network of similarities overlapping and criss-crossing” (Needham, 1975, p. 350). In the FRA model of NOS proposed by Irzik and Nola in the late 2000s, science was conceptualised as:

… a cognitive and social system whose investigative activities have a number of aims that it tries to achieve with the help of its methodologies, methodological rules, the system for knowledge certification and dissemination in line with its institutional social-ethical norms, and when successful, ultimately produces knowledge and serves society (Irzik & Nola, 2014, p. 1014).

The FRA is not merely a theoretical tool that is only focused on providing a philosophical account of what science is. Since the inception of the FRA as an approach to teaching about the NOS, practical suggestions on using the FRA in science education and science teacher education have been made by specifying the categories with respect to which resemblances can be found, and visualising the categories and their relations (Erduran & Dagher, 2014). The approach has since been used widely to analyse science curricula and textbooks (Caramaschi et al., 2021; Cheung, 2020; Park et al., 2020a, b), investigate teachers’ and students’ perceptions of the NOS (Peters-Burton et al., 2022; Wu & Erduran, 2022), and develop activities and interventions to teach various aspects of the NOS (Erduran & Kaya, 2019; Kaya et al., 2018, 2019).

Despite the fruitfulness of the family resemblance concept, in analytic philosophy, using it to define a concept has been faced with some challenges. Most notably, some critics have focused on the “wide-open texture” of family resemblance (Bellaimey, 1990; Williamson, 1994); that is, since we can always find similarities between instances of one concept and those of another, there is no limit to the extension of concepts (Andersen, 2000). When it comes to “science” as a family resemblance concept, the resemblance with a focus on “similarities” might eventually extend to certain activities that are not scientific but resemble science in some respects. This would be an undesirable conclusion for science educators, particularly given the increasing need for individuals to distinguish pseudoscientific claims circulated in the media from science (Höttecke & Allchin, 2020). The problem stems in part from the fact that family resemblance, as evident from its name, tends to foreground similarities that hold different branches of science together over what differentiates science from other activities. Some pseudoscientific and non-scientific activities resemble science with respect to their aim to explain nature, use of evidence-based methods, and interrelation with politics and society, as illustrated by the homoeopathy example. Hence, the crucial task is to extend the discussion about the FRA in a way that can account for not only how different sciences can be grouped together but also how science is distinct from pseudoscience.

Whilst NOS researchers have made substantial progress in explaining sciences, the notion of pseudoscience and how it relates to NOS frameworks remain largely understudied. How can science and pseudoscience be addressed coherently in science education? How can students be supported to critically analyse individual instances of pseudoscience (e.g., homoeopathy, astrology and climate change denial) as well as understand what ties these instances together?

In this article, we capitalise on the notion of family resemblance and the NOS to consider how the FRA can simultaneously explain the diversity and unity of science and support teaching about the nature of science and pseudoscience in science education. It aims to clarify how the problem of pseudoscience relates to the NOS in science education, compare the “consensus view” of the NOS and the FRA in their capacities for dealing with pseudoscience, and propose guiding principles for teaching about pseudoscience in schools. Before elaborating on the relation between the NOS and pseudoscience specifically, we first focus on the issue of pseudoscience in general to consider the need for tackling and teaching about it in schools and review existing evidence about pseudoscience in the context of science learning.

Our discussion in this article will be focused on, and limited to, pseudoscience defined as “a pretended or spurious science; a collection of related beliefs about the world mistakenly regarded as being based on scientific method or as having the status that scientific truths now have” (Oxford University Press, n.d.), as opposed to legitimate science. Defined as such, pseudoscience is also distinguished from “nonscience” (such as literary criticism and art theory), “bad science” (i.e., the results of legitimate but unsuccessful scientific activities) and “scientific fraud” (e.g., fabrication, plagiarism and deception), although these concepts sometimes may overlap with pseudoscience in some cases (see Hansson, 2021 for further discussion).

## Why is Pseudoscience Important in Science Education?

Recently, beliefs that contradict mainstream scientific knowledge have become particularly consequential because, in several areas, they may influence the behaviour of individuals in ways that harm themselves or others. For example, during the COVID-19 pandemic, vaccine hesitancy arising from erroneous beliefs about the origin or spread of the coronavirus has had significant public health consequences (Goldenberg, 2021). Climate change denial inhibits personal and collective action to mitigate an urgent global crisis (Oreskes & Conway, 2010; Stott, 2021). Favouring unproven or discredited medical treatments, such as homoeopathic or naturopathic medicine, can cause both individual and societal harm (Hermes, 2018; Smith, 2012). In some domains, such consequential heterodox beliefs are on the rise. For example, surveys report that in some countries including South Korea, Indonesia and Pakistan, confidence in vaccines fell in the period 2015–2019 (De Figueiredo et al., 2020) and the mid to late 2000s has seen growing international climate scepticism (Capstick et al., 2015). Such trends in beliefs with societal consequences suggest that addressing pseudoscientific beliefs should be an aim of science education.

Whilst mitigating belief in pseudoscience might seem an unproblematic aim of science education, there are several issues that need to be considered when developing pedagogical interventions. First, as we discuss below, how pseudoscience is defined and approaches to the specification of criteria for demarcating science from pseudoscience are contested aspects of research. If teachers are asked to support students’ identification of pseudoscience, they need to be given guidance on how to describe demarcation processes. In this article, we make some suggestions for how teachers can represent a complex categorisation process at an appropriate level of simplification for their students. Second, empirical research suggests that countering beliefs directly, for example, by presenting evidence that contradicts a claim, can fail to cause belief change (Fackler, 2021; Limón, 2001; Nickerson, 1998). People tend to selectively interpret and accept evidence in ways that allow them to maintain their existing beliefs (Corner et al., 2012). This cognitive bias suggests that care needs to be taken when developing pedagogical approaches to changing pseudoscientific beliefs, as some well-intentioned teaching approaches may reinforce the positions that the teacher is hoping to change. In the final section of this article, we consider plausible approaches to addressing pseudoscientific beliefs.

Teaching about pseudoscience in the science classroom might be thought of as having two goals. The first is the pragmatic aim of teaching students that certain activities are considered scientific or pseudoscientific by a consensus of experts. For example, students might be taught that the claim that mobile phone masts cause the transmission of viruses is a pseudoscientific belief. Such teaching can prevent harm, both to individuals and society, and encourage prosocial behaviours. The secondary, less immediately practical aim is to, by demarcating the nature of pseudoscience, support students’ understanding of the NOS and epistemology more generally (Erduran, 1995). That is, teaching can support students’ understanding of the process by which science can be demarcated from non-science (for example, by introducing the FRA) and their ability to identify pseudoscientific claims. How science and pseudoscience are demarcated, the process of demarcation, has implications for how the NOS (and that of pseudoscience) are understood and raises questions about who has the authority to make decisions about whether certain forms of knowledge, for example, traditional scientific knowledge, are categorised as scientific. Whilst less immediately practical than the first aim, the second goal might be imagined to support students to develop a transferable capacity to distinguish science and pseudoscience, across contexts, including novel practices or situations that have not been directly addressed in lessons. For example, the rise in the belief that mobile phone masts are responsible for the transmission of viruses is a relatively novel pseudoscientific belief that it would have been difficult for teachers to anticipate and so directly teach about. Teaching that supports students’ ability to transfer learning to novel contexts might prevent the development of certain types of pseudoscientific beliefs. For example, by considering the evidence that mobile phone signals cause cancer, a teacher might introduce substantive knowledge about microwaves, model a critical stance towards claims, and set out strategies for evaluating media reports that protect students from adopting erroneous beliefs. Teaching students about particular cases of pseudoscience is valuable, but supporting them to acquire an approach that can reliably demarcate science from pseudoscience across contexts might be considered a particularly valuable epistemic (and practical) achievement and hence a worthy goal of science education. Below, we discuss some approaches that have supported domain independent abilities, such as the ability to critically evaluate evidence, that may support the identification of pseudoscience.

Before considering pedagogic interventions to minimise belief in pseudoscience, it is worth considering studies that have reported the prevalence of pseudoscientific beliefs amongst students and science teachers. Only a handful of studies have sought to document the pseudoscientific beliefs of school-age students. A survey of 2159, 11-to-16-year-old students in England found that scepticism towards pseudoscience varied with gender, with male participants showing higher degrees of scepticism than female participants and that scepticism generally increased with age (Preece & Baxter, 2000). For example, whilst over three-quarters of female students aged 11 to 14 years old reported a belief in ghosts, only 39% of male 16–18-year-old students held the belief. Amongst 11–14-year-old students, 46% of female students believed in astrology and 54% in the negative impact of Friday the 13th. The study also found that nearly a third of 50 trainee science teachers surveyed believed in ghosts and a quarter in the healing power of crystals. Lundström and Jakobsson (2009) reported data from a survey of pseudoscientific beliefs related to health from a sample of nearly 300 upper-secondary students in Sweden. A high degree of belief was reported in the claim that acupuncture can relieve pain, the existence of telepathy and the effectiveness of magnetic bracelets in the treatment of arthritis, amongst other beliefs. The authors reported that students could score highly on assessments of scientific knowledge whilst retaining an adherence to pseudoscientific beliefs. A study of fourteen, roughly twelve-year-old, students in Turkey found a majority of the participants believed that crystals had medical powers (Metin et al., 2020). The students failed to activate scepticism that they acknowledged in other topics when claims aligned with their existing beliefs.

In addition to students’ ideas, the prevalence of pseudoscientific beliefs amongst science teachers will have consequences for the delivery of training on pedagogical approaches. Uçar and Sahin (2018) described the results of a survey of around 120 pre-service science teachers at a university in Turkey. They found that many respondents struggled to distinguish some scientific concepts from pseudoscientific beliefs. For example, members of the cohort believed that magnets could be used as a therapeutic treatment for certain conditions and that ghosts existed. Also in Turkey, Kaplan (2014) reported that most pre-service science teachers in a sample believed astrology to be a scientific practice, with some respondents arguing that astrologers’ adoption of “statistical” approaches made the activity scientific. By contrast, Turgut (2011) found that amongst a sample of around 50 pre-service science teachers in Turkey, whilst none of the respondents felt astrology was scientific, around 40% of the participants were uncertain as to the pseudoscientific status of the activity. Whilst the teachers surveyed were generally positive about the inclusion of explicit teaching about pseudoscience, the participants were divided over the use of exemplar cases of pseudoscience, such as astrology, in teaching. The majority of the teachers argued that students would encounter common examples of pseudoscience in their everyday lives, so they should be prepared for those encounters; a minority believed that the introduction of pseudoscientific concepts might lead to the acceptance of misconceptions in preference to scientific ideas. The participants reported feeling underprepared for teaching about the demarcation of science and pseudoscience. Fuertes-Prieto et al. (2020) reported that the prevalence of pseudoscientific beliefs (including beliefs in healers, paranormal phenomena and horoscopes) in a cohort of Spanish pre-service teachers was similar to, if not higher, than levels of belief in the general population. A survey of undergraduate education students in the USA (Losh & Nzekwe, 2011) found that whilst science teachers held fewer pseudoscientific beliefs than other teachers, some of the scientists believed in paranormal phenomena, including the occurrence of extra-terrestrial visits, and the existence of fantastic beasts (such as the Loch Ness Monster). Such findings suggest that novel interventions to minimise school students’ belief in pseudoscience should first ensure that teachers are confident in demarcating science and pseudoscience and that any pseudoscientific beliefs held by teachers that are related to content on the school science curriculum have been countered in professional development activities.

## Interventions to Mitigate Pseudoscientific Beliefs

Recent work on pseudoscience has tended to start from the demarcation problem, that is, the question of how science and non-science can be differentiated (Gordin, 2021; Pigliucci, 2013; Pigliucci & Boudry, 2013). Whilst there is an extensive research programme related to defining the NOS, and the most appropriate approaches to specifying that nature (Abd-El-Khalick & Lederman, 2000; Lederman, 2007; Matthews, 2012), there is no corresponding research programme debating the nature of pseudoscience. This omission is significant as Pigliucci (2013) has argued that thinking about “what X is” can be supported by indicating “what X is not”. A consideration of the nature of pseudoscience, and the manner in which it is demarcated from science, will support a clearer understanding of the NOS itself. Several authors have claimed that teaching about pseudoscience can inform understanding of the NOS, and the cases of dowsing (Afonso & Gilbert, 2010), and astrology (Turgut, 2011) have been used to teach the NOS. By contrast, Martin (1994) advocated for the explicit teaching of pseudoscientific topics, such as paranormal phenomena, in the science curriculum to highlight the nature of pseudoscience itself. He argues that pseudoscience beliefs often share surface features with scientific knowledge (for example, reference to empirical evidence and the use of technical language) but lack deep features such as falsifiability and their believers lack an openness to criticism. Cases such as astrology may be used to indicate this difference. Rather than conceptualising teaching about pseudoscience as an opportunity only to illustrate the NOS, we suggest teaching about the boundaries of the two concepts can be valuable for defining the nature of both classes of human activities.

If teachers are to introduce students to a general approach to demarcating pseudoscience from science in the classroom (and we have argued this strategy may bring additional value beyond a case-by-case demarcation), they would require guidance on how to present a demarcation that applies across contexts. A number of approaches to teaching about demarcation have been suggested. Fernandez-Beanato (2021) used the case of teaching about feng shui to argue that demarcation should occur via the proposal of a list of defining features of science. The challenge of producing a definitive list of characteristics of science and pseudoscience has been noted by a number of critics (Hansson, 2013; Pigliucci, 2013). An alternative approach, proposed by Bhakthavatsalam and Sun (2021), demarcates science from pseudoscience through a virtue epistemological approach. Bhakthavatsalam and Sun argue that science is associated with several epistemic virtues, for example, those practices which lead to the establishment of reliable knowledge (critical thought, evaluation of sources, etc.), which do not occur in the practice of pseudosciences. However, the epistemic virtue approach suffers from the same critique as list-based approaches to demarcation—it is possible to propose examples of scientific and pseudoscientific activities that are not covered by descriptions of epistemic virtues.

In the context of higher education, several quasi-experimental studies have evaluated approaches to reducing adherence to pseudoscience through fostering domain-independent abilities, for example, by developing critical thinking (Adam & Manson, 2014; McLean & Miller, 2010; Wilson, 2018) and supporting students’ argumentation (Tsai et al., 2015). Turgut (2011) investigated whether considering demarcation in a single context, the case of astrology, would develop the NOS understanding of individuals enrolled in a teacher education course. Results indicated that the instructional strategy was effective to some extent, and a majority of the teacher candidates who participated in the research reported they would use pseudoscientific cases in their teaching of the NOS. In another context-situated approach, Afonso and Gilbert (2010) found that a small number of Portuguese undergraduate students judged water dowsing to be a scientific practice. When asked to develop a test for the effectiveness of the dowsing, the authors argue belief bias, the tendency to avoid searching for alternative explanations to committed positions, led the students to develop invalid tests for the phenomenon. The research programme focused on interventions to mitigate against pseudoscientific beliefs is insufficiently advanced to present reliable conclusions about whether case-focused or more general strategies are preferred on empirical grounds.

## Why is Pseudoscience Difficult to Teach?

Whilst, for the reasons discussed above, it is desirable to minimise students’ adherence to pseudoscientific beliefs, and in particular those with societal consequences (for example, beliefs in the inefficacy or harmful nature of vaccines), three factors make this aim difficult to achieve. First, several studies have reported that, despite expectations, greater levels of scientific knowledge do not correlate with lower adherence to pseudoscientific positions (Eve & Dunn, 1990; Johnson & Pigliucci, 2004; Walker & Hoekstra, 2002). Simply increasing students’ scientific knowledge bases does not automatically lead to a reduction in belief in pseudoscience. Such findings suggest that there is value in teaching about pseudoscience itself. Introducing people to evidence that contradicts their beliefs, it has been claimed, may only strengthen their commitment to those positions (Nyhan & Reifler, 2010), by the backfire effect. However, the data supporting the backfire effect is not robust and the existence of the phenomenon remains a matter of debate (Swire-Thompson et al., 2020). Whilst the extent to which discordant information can strengthen existing beliefs is unreliable, there is much empirical support for confirmation bias, a tendency to interpret information in a manner that preserves pre-existing beliefs (Nickerson, 1998). An aspect of confirmation bias is the attitude polarisation effect in which discussion of a topic can lead to positions becoming more extreme (Isenberg, 1986). A large body of research on causing change to substantive scientific concepts (such as beliefs about forces) suggests that inducing conceptual changes through the introduction of evidence that contrasts with existing beliefs are rarely successful (Limón, 2001). Such features of cognitive systems make minimising pseudoscientific beliefs challenging.

The second difficulty arises from a consensus amongst philosophers of science that demarcating science and pseudoscience is challenging and that it may be impossible to specify a list of necessary and sufficient conditions that define membership in the group of scientific or pseudoscientific beliefs or activities (Cleland & Brindell, 2013; Law, 2020; Pigliucci, 2013). The lack of consensus demarcation criteria makes directly teaching the distinction between science and pseudoscience difficult. The absence of demarcation criteria may arise because judgements of the scientific or non-scientific status of claims and activities draw on tacit knowledge (Hansson, 2013). Whilst it can be difficult to report explicit demarcation criteria, it is accepted that experts can often intuitively recognise activities as scientific or pseudoscientific, implying that they are drawing on inarticulable (i.e., tacit) knowledge (Pigliucci, 2013). Whilst tacit knowledge is an element of scientific expertise and its appropriate development is significant, particularly in domains that draw heavily on embodied experiences, such as forces and dynamics (Brock, 2015), in the context of teaching about pseudoscience, a lack of explicit, and therefore directly teachable criteria present a challenge for teachers. Third, a student’s cultural and family environment can reinforce and so make the revision of pseudoscientific beliefs challenging (Lobato & Zimmerman, 2018; Soltani, 2020). Pseudoscientific ideas can be elements of belief systems to which people have strong commitments and revisions may, in some cases, require a significant change in viewpoint which can be challenging and so resisted. For example, a belief about the age of the Earth may be part of a system of beliefs, including both empirically testable claims and metaphysical beliefs.

## Pseudoscience From Two Approaches to NOS

Although there is a general awareness that the nature of pseudoscience is closely related to the NOS in science education (Allchin, 2013; Matthews, 2019), there has been little discussion around how pseudoscience can be understood in relation to existing NOS frameworks. In this section, we will consider how pseudoscience can be understood from two major conceptualisations of the NOS, namely, the consensus view and the family resemblance approach. Compared to substantive scientific content knowledge, the topic of the NOS is fraught with controversy, particularly with regard to how to conceptualise the NOS and what to teach about it (Dagher & Erduran, 2017; Matthews, 2012; Schwartz et al., 2012). Although the consensus view and the FRA can be compared from multiple angles, this section will focus on their potential for addressing the demarcation problem and pseudoscience in science education.

### The Consensus View and Pseudoscience

The “consensus” view of the NOS holds that there exist some general aspects of the NOS that are agreeable to relevant experts and can be taught to students (Lederman et al., 2002, 2014a, b; McComas, 1998). According to one popular version of the consensus view, these aspects include the empirical NOS, creativity and imagination in science, scientific laws and theories, observations and inferences, the social and cultural embeddedness of science, the subjective NOS and the tentative NOS (Lederman, 2007). The main strength of the consensus view is that, since it consists of tenets that can hardly be objected to, it provides a concise and systematic conception of NOS that can make it easy to teach in the classroom. However, the consensus view has been criticised on several fronts. For example, the very assumption that there is a consensus on some non-trivial features of science on a certain level of generality has been questioned (Hodson, 2014; Irzik & Nola, 2011; Matthews, 2012). Other aspects of the consensus view, such as the separation of scientific knowledge and scientific inquiry, have also been contested (Hodson & Wong, 2014). For the purpose of this paper, the most critical issue with the consensus view is that it risks obscuring the disunity and heterogeneity of science by focusing on “general” statements that are declarative and universal (van Dijk, 2011). This is because it is hard to group instances of science together in terms of general features shared amongst all the instances, and the same difficulty arises for instances of pseudoscience, as will be discussed later.

Although there can be benefits to discussing some representative characteristics of pseudoscience, the key point is that we should not take these as complete demarcation criteria but as aspects of pseudoscience that could be discussed and critically reflected on. Recent approaches to demarcation tend to specify multiple criteria rather than one. Hansson (2021) summarises seven key criteria of pseudoscience commonly found in the lists proposed by philosophers of science:

1. Belief in authority: Some individuals have the authority to decide what is true or not.
2. Unrepeatable experiments: Experiments that cannot be repeated by others are believed.
3. Handpicked examples: Examples that are not representative of a category are used to support claims.
4. Unwillingness to test: There are no endeavours to test a theory even when it is possible.
5. Disregard of refuting information: Only evidence and examples that support a theory are emphasised.
6. Built-in subterfuge: Testing is designed in a way that can only confirm the theory.
7. Explanations are abandoned without replacement: Explanations that worked are discarded without being replaced, leaving much more unexplained by the new theory than the old theory.

There is a strong similarity between the consensus, tenet-based conceptualisation of NOS and the early approaches to demarcation in philosophy of science which sought to establish a set of criteria for identifying pseudoscientific practices (Pigliucci, 2013). The consensus view researchers admit that the lists are insufficient and incomplete (Schwartz et al., 2012) but consider that they can still provide aspects of the NOS that are developmentally appropriate and little disputed at some level of generality (Lederman et al., 2014a, b). What makes the consensus view problematic as a potential lens for pseudoscience (although, to our knowledge, no published study has directly taken on this task), in our view, is its focus on “consensus”. The consensus-based approach can have limited capacity in addressing the demarcation of pseudoscience, where even less consensus exists compared to in the context of defining science. However, the impossibility of establishing a definitive set of demarcation criteria need not imply that we cannot or should not teach about pseudoscience in schools. As Pigliucci (2013) rightly expressed, “even if we do not arrive at a neat and exceptionless formal definition of some X, based on a small set of necessary and jointly sufficient conditions, we may still come to learn a lot in the process” (p. 2). Besides, using the consensus approach to address pseudoscience can also be challenging because, similar to when applied to science, there are too many variations across different (pseudo)scientific fields to be captured by a single set of characteristics.

Also note that each of the aspects in Hansson’s list above is closely tied to the aspects of the NOS. Let us consider “unwillingness to test” (although it is possible to test) as a major feature of pseudoscience. This may be true for some instances of pseudoscience but not for others; proponents and practitioners of homoeopathy and alternative medicine are not opposed to testing the effect of the remedies. Given that “test” in this case is a loaded term, the homoeopathy example provides an opportunity for students to discuss what it means to “test” hypotheses and theories in science. Similarly, Hansson (2021) refers to “handpicked examples” as a problematic feature of pseudoscience. This practice has been particularly evident in the case of climate change denial and the “manufactured” debate about the health effect of tobacco products (Oreskes & Conway, 2010). It should be noted, however, that the cherry-picking of favourable evidence and the concealment of unfavourable evidence can and does happen in legitimate sciences, too. The growing debate around so-called p-hacking, where researchers relentlessly search for groups, cases and categories that show statistically significant differences in terms of some variables (Head et al., 2015), is a vivid example of such cherry-picking. This example suggests that a list-based approach to science and pseudoscience can be misleading and oversimplify the complexity of the issue, calling for an alternative approach.

### FRA and Pseudoscience

The greatest success of the FRA thus far has been in its ability to explain how different “sciences” can be bound together as a single concept without having to neglect the specificities of individual scientific domains. Irzik and Nola (2014) demonstrated how scientific disciplines such as astronomy, particle physics, earthquake science and medicine can constitute a family even though there are characteristics not shared by some of these instances (e.g., astronomy lacks hypothesis testing; earthquake science lacks experimentation). In this way, the FRA can explain the diversity and heterogeneity within science whilst maintaining the unity of the concept (Irzik & Nola, 2014). Erduran and Dagher’s (2014) extended FRA takes a step further to conceptualise the NOS in terms of eleven categories grouped into three “wheels”. The wheels and categories have served as an effective tool to examine a broad range of NOS characteristics in a nuanced and comprehensive manner (Erduran & Dagher, 2014). Contrary to this focus on similarity, what has been much less articulated is whether and how the FRA could be used to address the *dissimilarity* between science and what is not science. We believe that this issue around similarity and dissimilarity is not only philosophical but also psychological, that is, closely related to how humans learn about abstract concepts, a relation that is crucial in developing pedagogical implications for teaching about pseudoscience, as will be elaborated later in this section.

A critical difference between the consensus view and the FRA lies in what each approach aims to provide for NOS instruction. The consensus view aims to provide the content of the NOS by listing salient features of science. The FRA has a different sort of aim. The eleven categories organised into three “FRA wheels” (Erduran & Dagher, 2014) are not intended as answers (whether complete or not) to “what science is”. Instead, these provide a framework for thinking about NOS as well as selecting, organising and presenting the NOS in curriculum and instruction. For this reason, some have acknowledged the compatibility between consensus NOS tenets and FRA categories (Irzik & Nola, in press; Kampourakis, 2016).

The family resemblance characterisation of a concept (e.g. game) is in opposition to essentialist definitions (e.g. a “triangle” being a plane figure with three sides and three angles, and such a plane figure being a triangle; see Irzik & Nola, in press, for a detailed discussion). In this regard, there seems to be little doubt about the assumption that “science” and “pseudoscience” are family resemblance concepts (Pigliucci, 2013). It follows that the task of defining science and pseudoscience is significantly different from defining concepts like a triangle, a bachelor or a vertebrate. For science, even the advocates of the consensus view acknowledge that a list-based approach is not intended to be *philosophically* seamless; it is primarily the *pedagogical* considerations (e.g., developmental appropriateness) where their strongest rationale comes from (Lederman et al., 2014a, b; McComas, 2020). For pseudoscience, the broad agreement amongst philosophers of science that there is no definitive set of demarcation criteria supports that pseudoscience is a family resemblance, rather than an essentialist, concept. In terms of addressing pseudoscience, the FRA has relative strength in that it does not *prescribe* any characteristics of science with regard to the categories. However, it may seem that the FRA rejects the existence of any criteria for science and therefore implies that anything that “resembles” science can eventually be considered as a science. In our view, the current discussion of the FRA is focused on how different fields can be collectively called “sciences” rather than how they can be distinguished from what is not science. This problem is related to the fact that pseudosciences pose as legitimate science and therefore often resemble legitimate science in terms of epistemic and social features (Mahner, 2013). Matthews (2019) elaborated on this point in his book on the specific case of feng shui (Chinese geomancy):

All pseudoscience contains some scientific content – concepts, mathematics, instruments, and measurements – in order to give the practice credibility. It is of the essence of pseudoscience to appear to be scientific; its ‘authority’ depends on mimicking science. Science has journals, so pseudosciences commence their own or “take over” established journals; science has peer review, so pseudoscience has the same; science has numbers and statistics, so pseudoscience has tables, figures, and correlations; science has experiments, so pseudoscientists conduct their own; and science has meetings and conferences, so pseudoscience does the same. (p. 270)

One thing that we can infer from the FRA for teaching about pseudoscience is that instances of pseudoscience should be considered on a case-by-case basis (Irzik & Nola, 2011). Proponents of the FRA argue that when teaching about the NOS, rather than applying one-size-fits-all criteria about what science is, each case should be considered in relation to other examples of sciences (Erduran & Dagher, 2014; Irzik & Nola, 2011). Likewise, in considering different instances of pseudoscience, it is vital that learners are given the opportunity to examine the similarities as well as dissimilarities between instances of pseudoscience and between instances of science and those of pseudoscience.

Whilst most existing work has focused on family resemblance as an account of the concept of “science”, family resemblance has also been utilised by philosophers to explain the process of *learning* different natural kinds such as science, most notably by Kuhn (1974) and Andersen (2000). Kuhn famously argued that normal science progresses through scientists’ recognition of the similarity between “exemplars”, that is, a set of puzzles and solutions and a new set of puzzles and solutions within the paradigm, rather than through explicit rules (Kuhn, 1974). He draws on Wittgenstein’s concept of family resemblance to explain this similarity. In an essay titled *Second Thoughts on Paradigms*, Kuhn uses a metaphor of a child learning to identify swans, geese and ducks to describe how scientists gain knowledge through a grasp of family resemblance relations. In this hypothetical account, an adult who is familiar with these classifications guides the child through a series of ostensive acts as the child grasps how to distinguish these birds. The child is shown various instances of these birds and the adult tells him what category each instance belongs to. The child is also encouraged to point out instances of each category. Through this process, the child gradually acquires competency to distinguish and identify the birds. Kuhn states that “anyone who has taught a child under such circumstances knows that the primary pedagogic tool is ostension. Phrases like ‘all swans are white’ may play a role, but they need not” (p. 309). He also says that “The resultant ability to see a variety of situations as like each other is, I think, the main thing a student acquires by doing exemplary problems, whether with a pencil and paper or in a well-designed laboratory” (p. 306).

Learning about science and pseudoscience can occur in a similar manner to that Kuhn described. As discussed earlier, the ability to differentiate legitimate sciences and pseudoscience pertains, in part, to a grasp of tacit, rather than explicit, knowledge (Ladyman, 2013). For this reason, Kuhn’s account of learning by recognising similarities and dissimilarities can be powerful for cultivating students’ tacit knowledge relating to pseudoscience. Teaching through exposure to a range of cases of pseudoscience, without an expectation of the statement of definitive rules of category membership (though some suggestions, as in Hansson’s list, can be useful) might be thought of as an example of implicit learning (Reber, 1989). From the perspective of the FRA, learning about why astronomy, geology, particle physics and organic chemistry are scientific despite their differences is similar to learning to distinguish between swans, geese and ducks; Learning about how astronomy is different from astrology is similar to learning to differentiate birds of prey from waterfowl. The point is that this process is far from establishing explicit rules, criteria or algorithms to sort the instances into different categories. This use of family resemblance as an account of learning has not drawn much attention within the science education community (with the exception of Andersen, 2000), but there is evidence from psychological research that human learning of categories can be described as the acquisition of family resemblances based on exemplars (Rosch & Mervis, 1975; Smith & Medin, 1982; Ward & Scott, 1987). Although these psychological experiments were for learning concrete concepts such as “birds” and “furniture”, rather than human activities such as “science”, there seems to be some parallel that can be drawn.

To summarise the discussion in this section, we can conclude that (a) “pseudoscience” is a family resemblance concept for which no definitive set of demarcation criteria can be established; (b) current FRA models have concentrated on the similarities between sciences rather than what distinguishes science from pseudoscience; and (c) there is philosophical and psychological evidence that supports the possibility of learning complex concepts through recognition of family resemblances. Based on these ideas, in the following section, we will illustrate how we can use the FRA to teach about science and pseudoscience, whilst addressing the challenges of teaching about pseudoscience and evading the risk of the “wide-open” texture of family resemblance concepts.

## Principles for Teaching About Pseudoscience Through Family Resemblance

Given the inherent impossibility of generating a complete list of demarcation criteria, a more realistic and modest goal for us would be to establish some principles that can guide curriculum design and instruction when using FRA to teach about pseudoscience. Based on the discussion in the previous sections, we suggest three principles for teaching about pseudoscience at the secondary school level.

The first principle is that pseudoscience needs to be taught in a contextualised manner. Contextualised learning involves the use of various contexts (e.g. historical episodes and records, student-led activities, socioscientific issues) for learning pseudoscience. When teaching about pseudoscience, the focus on context includes understanding the key claims of a pseudoscientific field and the evidence used to support such claims and, based on such information, understanding why the claims might *seem* plausible and scientific (e.g., the institutionalised aspects of homoeopathy). We want students to be critical of pseudoscience, but a critical attitude should not consist in an outright rejection of claims without careful examination. As Bhakthavatsalam (2019) rightly noted, false beliefs, ideas and theories can be educationally beneficial when properly utilised. To do this, teachers may use documentary films that critically examine instances of pseudoscience, such as *An Inconvenient Truth* (2006) about climate change, *Behind the Curve* (2018) about flat Earth beliefs or *The Anti-Vax Conspiracy* (2021) about the anti-vaccination movement. These documentaries investigate the people and groups behind these pseudoscientific beliefs, their motivations and the “evidence” they use to support their beliefs. Students can then be introduced to documentaries and other resources focused on the practices of legitimate science (e.g., black hole detection, vaccine development, space science) and asked to compare the examples in terms of the FRA categories. In doing so, it is essential to recognise that learning about NOS would not automatically lead to understanding what science is *not*, although the two are mutually related. As discussed earlier, a key to avoiding the problem of “similarity” infinitely extending is to consider dissimilarity between the examples of science and those of pseudoscience. Based on the FRA, the latter would need separate instructional intervention focused on dissimilarities grasped from the comparison of exemplars. When developing contextualised approaches to teaching about pseudoscience, teachers should carefully consider the information that they make available to students that ground the cases introduced, for example, how much historical or social context is necessary to make an informed judgement.

The second key principle that can be drawn is that teaching about pseudoscience should be based on a “case-by-case” approach where a range of examples of pseudoscience is analysed, criticised and then reflected upon. This approach contrasts with constructing a list of features that define pseudoscience (or conversely, a list for science) to teach about what pseudoscience is and what it is not. Concepts such as “triangle” can be defined in terms of necessary and sufficient conditions, but neither “science” nor “pseudoscience” can be properly defined this way. Such a case-by-case approach is not only philosophically adequate but also supported by evidence from developmental psychology about how people learn concepts (Rosch & Mervis, 1975; Smith & Medin, 1982; Ward & Scott, 1987). Teachers should reflect carefully on their choice of cases as the selection will emphasise or minimise particular features of pseudoscience. From a family resemblance perspective, such case-based instruction does not mean that all that students would take home from the activity would be knowledge of individual instances of pseudoscience. Through engagement with actual cases, students can also develop explicit and tacit knowledge relating to the meta-criteria that differentiates pseudoscience from sciences by grasping these “concepts” that are connected by family resemblances.

Let us use the homoeopathy example to illustrate these two principles. Students can search the internet to see what the claims of homoeopathy are and the way practitioners justify its effectiveness. Once they have an idea of what it consists of, they can be introduced to other instances of alternative medicine (e.g., acupuncture, chiropractic) to analyse the similarities and differences with respect to the FRA categories, such as hypothesis generation or empirical testing. Through this activity, students will gradually recognise how the instances of pseudosciences resemble each other although they do not share the exact same set of features. Then, these instances can be compared with mainstream medicine, also on the basis of the FRA categories, to identify the dissimilarities. In this process, the teacher’s role is to facilitate these analytical discussions to cultivate family resemblance conceptions of science and pseudoscience. Specifically, the teacher needs to make the similarities and dissimilarities visible so that her students not only understand the individual similarities and dissimilarities but also gain a sort of metacognitive understanding of how they learned what they learned. In this way, students’ learning about the specific examples can be achieved explicitly although their knowledge of “science” and “pseudoscience” as family resemblance concepts will still be largely tacit.

Third, we suggest that the use of typologies and meta-criteria can help teachers facilitate systematic discussion about science and pseudoscience. This is because different types of pseudosciences can shed light on different FRA categories. FRA offers a lens to look into the similarities and dissimilarities between examples of legitimate sciences and pseudosciences, and between different branches of pseudoscience. In making these comparisons, typologies of pseudoscience can be utilised to draw attention to the diversity of pseudoscientific beliefs and activities and to explicate specific aspects of pseudosciences. Consider Gordin’s (2021) typology, where he classified instances of pseudoscience into four “families”: vestigial sciences, hyperpoliticised sciences, counterestablishment sciences and parapsychological sciences (Table 1).

**Table 1:** Typology of pseudoscience (based on Gordin, 2021)

	Description	Examples	Related FRA category
Vestigial sciences	Theories and beliefs that once counted as science but were rejected, so that they have morphed today into being classified as pseudoscientific	Astrology Alchemy Feng shui	Knowledge Social certification and dissemination
Hyperpoliticised sciences	A set of positions closely affiliated with repressive political regimes	Aryan physics Lycenkoism Eugenics	Political power structures Social organisations and interactions Financial systems Scientific ethos Social values
Counterestablishment sciences	Pseudosciences that are defined in opposition to professional scientists and mimic the structure of mainstream science	Phrenology Creationism Cryptozoology Cosmic catastrophism UFOlogy Flat Earth	Social certification and dissemination Professional activities Social organisations and interactions
Parapsychological sciences	Theories and beliefs in the powers of mind that extend beyond the canonically recognised five senses of sight, hearing, smell, taste and touch	Parapsychology	Methods Practices Knowledge

A complete review of these types of pseudosciences is besides our goal, but it is useful to note that these types are not intended to be exhaustive or fully exclusive to each other (Gordin, 2021); nor do we argue that the examples within each type all share the same characteristics in terms of the FRA categories. Examples in each of these categories can be used to highlight how pseudosciences are similar to legitimate sciences with respect to some (but not all) of the eleven FRA categories. Similarly, on feng shui, Matthews (2019) emphasises that feng shui as it is practised today has all the aspects of NOS but only as “simulacra”:

The key elements of science—content, methodology, experiment, mathematization, theoretical and conceptual growth and refinement, a scientific habit of mind, and social organization—are present only as simulacrums. There is no tradition of the controlled and reproducible experiments; there is no recognition of the defect of ad hoc rescuing of failed hypotheses; there is no effort to disentangle variables and study their contributions; there is a dramatic inconsistency with the core of established scientific knowledge, most especially the conservation of energy postulate; there is no effort to explain this inconsistency by engagement with the scientific community; there are no contributions to established, peer-reviewed, scientific research journals. (pp. 293–294).

In a book-length study of feng shui from the perspective of science education, Matthews (2019) presents a helpful way of engaging with pseudoscience that, with some scaffolding, can be utilised for resemblance-based instruction. He first examines the theoretical components of feng shui and the accounts of nature and the world that it provides, and then traces the historical and cultural origins of feng shui and its current scientific status. He scrutinises the concept of “qi” which is central to feng shui and compares it with “energy” and argues that feng shui has some similarities with science in terms of its epistemic and social structures, but there are important differences. In the science classroom, students can be invited to review feng shui resources, identify major claims and evidence, and discuss the resemblances with legitimate science with respect to the FRA categories. The teacher can prompt this process by asking, for example, “What are some inconsistencies between feng shui claims and established scientific knowledge? How have feng shui advocates responded to such inconsistencies? How is it similar or different to what scientists do when inconsistencies arise?”. These questions can create an opportunity to discuss core aspects of the NOS such as the growth and refinement of scientific knowledge, as well as the social process through which scientific knowledge is certified and accepted. Through this process based on the analysis of resemblances (i.e., similarities *and* dissimilarities), students can grasp not only *that* feng shui is a pseudoscience, but also *why* it is one and develop general skills transferrable to other situations.

**Fig. 1:**
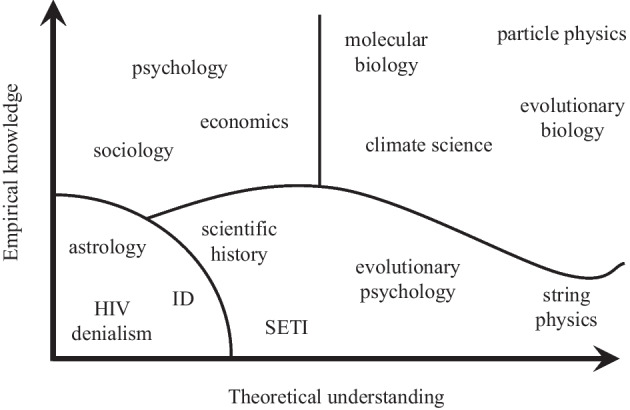
A taxonomy of science and pseudoscience in terms of theoretical understanding and empirical knowledge (Pigliucci, 2013, p. 23). The four sections represent different Wittgensteinian family categories: established sciences (upper right), soft sciences (upper left), pseudosciences (lower left) and proto-/quasi-sciences (lower right)

As such, the eleven FRA categories that encompass the epistemic and social characteristics of science can serve as helpful meta-criteria for analysing pseudosciences. In addition to Gordin’s taxonomy, Pigliucci’s two-dimensional taxonomy of sciences and pseudosciences (Fig. 1) can be helpful too for grasping the family resemblances between pseudosciences, particularly with respect to the “scientific knowledge” category in FRA. Pigliucci criticises the approaches to demarcation based on only one criterion (e.g., falsification) and instead suggests a two-dimensional model that takes into account both the theoretical and empirical knowledge bases of a field. The two-dimensional model has the affordance of not requiring the precise location of an activity against the axes; it can be indicated by a diffuse area, which coheres with the family resemblance approach. In a practical example, a teacher might print out Pigliucci’s two axes on pieces of blank paper and ask her students to draw in areas that represent, for example, psychology, vaccine denial and homoeopathy. A potentially fruitful follow-up activity would see students comparing their diagrams with other students and discussing differences in their location of activities. Alternatively, students could compare their categorisations with Pigliucci’s, below, and debate any discrepancies.

## Conclusions

The question of what science is underlies various fundamental issues within science education, especially at a time of anxiety about misinformation, conspiracy theories and fake news. In this article, our goal was to make a case that the NOS can play a central role in addressing pseudoscience in the science classroom and examine the implications of the two views of NOS for teaching pseudoscience. One of the key motivations for NOS instruction has been preventing an absolute and static image of science as an established body of knowledge and instead portraying it as a dynamic, tentative, social and cultural human activity. Although this approach is coherent with how historians and sociologists of science understand science today, for educators, there are some unanswered questions as to how we could help students recognise the difference between science and other human activities, some of which are disguised as legitimate science. We argued that there is currently limited discussion, from either the consensus view or the FRA, on how NOS approaches can address pseudoscience and the demarcation problem. We analysed how the consensus view and the FRA can each be related to these issues and demonstrated their respective strengths and limitations. As long as useful implications for practice can be drawn from such comparisons, they should not be viewed as unproductive or irrelevant topics for educators. That is, the focus of teaching about pseudoscience should not be on the demarcation itself (i.e., enabling students to call something science or pseudoscience), but rather on the various aspects of science that can be discussed in relation to demarcation through the process. When Laudan proclaimed the “demise of the demarcation problem” (Laudan, 1983), he was wrong in suggesting that discussing the demarcation problem was unproductive; Yet he was right in the sense that demarcation could be unproductive if the sole focus is to demarcate science from what it is not.

Considering FRA both as an approach to defining concepts such as “science” and as a theory of conceptual learning, the three principles will be useful in guiding future practice and research on the topic. For example, the principles can be applied to the contemporary issue of climate change denial, which is a form of pseudoscience (Hansson, 2017) and therefore can be contrasted with mainstream climate science. Philosophers have not been successful in finding what demarcates science and pseudoscience, and the task is unlikely to be achieved in the future, but discussing this issue has led to many important ideas about science. Popper’s interest in the difference between psychoanalysis and general relativity gave birth to the idea of falsification, which later turned out to be incomplete but is still an insightful idea. Students need to be able to analyse and critically assess instances of pseudoscience in different respects whenever they encounter them, and this should be the aim of teaching about pseudoscience. We do not want to simply give them a list of pseudosciences and tell them to avoid these; Nor do we want to create a checklist as a “litmus test” for distinguishing science and pseudoscience. Learning about pseudoscience could be more effectively achieved when based on the idea of family resemblance, by inviting students to explore, debate and reflect on diverse examples of scientific and pseudoscientific activities in the light of the eleven FRA categories. In doing so, we will develop students’ demaractive abilities, a valuable possession in the contemporary world.

We believe that our analysis and suggestions provide helpful guidance for teachers and researchers who wish to address pseudoscience in science education. The FRA provides a coherent framework that can be applied to critically analyse not only sciences but also pseudosciences. With a focus on similarities and dissimilarities, it enables teachers to discuss pseudoscience in the science classroom without purporting to present a checklist for demarcating science and pseudoscience. It can support both the explicit learning about the features of specific pseudosciences and the tacit learning that is transferrable and can be applied to novel situations that students encounter in their everyday lives. We note that our principles are only an initial step to combatting pseudoscientific beliefs that are becoming stronger and more harmful. Future research, by ourselves and hopefully others, needs to investigate the effect of different combinations of pseudosciences to facilitate students’ resemblance-based understanding, the types of instructional resources and interventions that can effectively mitigate pseudoscientific beliefs, and the relationship between individuals’ NOS understanding and their ability to discern pseudoscience from legitimate science.
